# Assessing the cardioprotective effect of necrosulfonamide in doxorubicin-induced cardiotoxicity in mice

**DOI:** 10.25122/jml-2023-0091

**Published:** 2023-10

**Authors:** Shaymaa Fadhil Abbas, Hussein Abdulkadim, Najah Rayish Hadi

**Affiliations:** 1 Department of Pharmacology, College of Medicine, University of Basrah, Basrah, Iraq; 2 Department of Pharmacology, College of Medicine, University of Kufa, Najaf, Iraq

**Keywords:** necrosulfonamide, acute doxorubicin cardiotoxicity, cardiotoxicity, myocardial lesion

## Abstract

This study aimed to determine the cardioprotective effect of necrosulfonamide (NSA), a pyroptosis and necroptosis inhibitor, against acute doxorubicin cardiotoxicity. Fifteen male mice were divided into three groups (n=5/group). Cardiotoxicity was induced by a single intraperitoneal injection of 20 mg/kg of DOX on the 3^rd^ day of the experiment. The control group received daily intraperitoneal (i.p.) injections of 5% DMSO for five consecutive days. The second group, the DOX group, received a single i.p. injection of 20 mg/kg DOX on the third day of the experiment. The third group, the DOX plus necrosulfonamide (NSA) group, received DOX injections like the second group and 5 mg/kg of NSA i.p. daily for five days, starting two days before the DOX injection. At the end of the study, animals were euthanized, and blood and tissue samples were collected. Various parameters, including cardiac troponin I (cTnI), TNF-α, IL-1β, caspase-1, glutathione peroxidase-4 (GPX-4), and hemeoxygenase 1 (Hmox-1), were measured using ELISA. Cardiac expression of the NF-κB gene was determined by RT-qPCR. A histopathological assessment of myocardial lesions was also performed. DOX administration significantly increased serum cTnI levels and tissue inflammatory biomarkers (TNF-α, IL-1β, caspase-1) while reducing tissue antioxidant enzymes (GPX-4, Hmox-1). In addition, it significantly increased nuclear factor-κB (NF-κB) gene expression compared to the control (about 10.5-fold elevation). Histopathological analysis revealed marked vacuolization and necrosis. However, pretreatment with NSA dramatically altered these findings, with serum cTnI levels significantly lower in this group compared to DOX. Inflammatory indicators decreased, and antioxidant enzymes were restored to varying degrees. NSA pretreatment downregulated NF-κβ gene expression and preserved near-normal myocardial morphology. Our results showed that NSA protected against DOX-induced cardiotoxicity, an effect likely mediated by its anti-pyroptotic, anti-necroptotic, and antioxidant properties.

## INTRODUCTION

Doxorubicin (DOX) is a powerful and indispensable chemotherapeutic drug, and one of its well-known adverse effects is cardiotoxicity. Despite extensive research into the mechanisms of cardiotoxicity, the molecular genesis of this disease remains unknown [1]. Cancer is one of the global leading causes of death, and its prevalence is increasing at an alarming rate [2]. Despite significant advances in modern oncotherapy, conventional chemotherapeutic agents, such as DOX, remain critical components of chemotherapeutic combinations used to treat hematological and solid cancers. Nonetheless, adverse effects such as acute or chronic cardiotoxicity may limit DOX use [2]. Although the exact pathophysiology of DOX-induced cardiotoxicity (DIC) is unknown, it is believed that oxidative stress, activation of pro-inflammatory signaling, apoptosis, as well as autophagy dysregulation all play a role.

Furthermore, novel mechanisms of DIC have recently been identified, including pyroptosis and necroptosis, which are recognized as forms of regulated cell death (RCD) pathways [3]. Pyroptosis is a caspase-1-dependent inflammatory cell death pathway. Caspase-1 is recruited and activated by the inflammasome, directing downstream pyroptosis and the maturation of proinflammatory cytokines IL-1β and IL-18. Gasdermin D (GSDMD) pore-forming activity was discovered to be essential for cell death and promoting IL-1β and IL-18 secretion during pyroptosis [3]. Necroptosis is another type of RCD triggered by immune ligands such as tumor necrosis factor (TNF) and Fas, which activates receptor-interacting protein kinases 1/3 (RIPK1/ RIPK3), which then activates the mixed-lineage kinase domain-like protein (MLKL1). Phosphorylated MLKL1 disrupts cell integrity by translocating to the inner leaflet of the plasma membrane [3]. The inhibitor necrosulfonamide (NSA) was recently identified, targeting both necroptosis and pyroptosis pathways. It blocks MLKL1 phosphorylation and prevents necrose-downstream effectors interactions [4]. It was also discovered to inhibit pyroptosis by interfering with GSDMD function and alkylating a key cysteine residue (Cys191 in humans or Cys192 in mice), thereby interfering with the N-terminal domain's ability to oligomerizeand form membrane pores [5]. Several preclinical studies reported that NSA mitigated inflammation in various organs and tissues through its inhibitory effects on necroptosis and pyroptosis pathways [6-8]. Given the protective benefits of NSA across several pathological processes, this study aimed to examine its protective effects against acute doxorubicin-induced cardiotoxicity.

## MATERIAL AND METHODS

Fifteen adult male BALB/c mice weighing 25 and 30 grams were purchased from the Faculty of Science at the University of Kufa. The animals were housed in a climate-controlled environment at 25±2 ^0^C and 12-hour dark/light cycles. The animals had unlimited access to water and standard food. They were acclimatized at the animal house for two weeks before being used in the experiments. The research followed the Institutional Guidelines for the Care and Use of Laboratory Animals. The High Committee approved all protocols for Review and Approval of Research Proposals, Faculty of Medicine, University of Kufa. DOX, HCl, and NSA were obtained from Beijing Jin Ming Biotechnology, China.

The mice were divided into three groups, with five mice in each group:

1. Control group: Mice in this group received daily intraperitoneal (i.p.) injections of 5% DMSO for five consecutive days.
2. DOX group: These mice received a single i.p. injection of 20 mg/kg DOX on the third day of the experiment.
3. DOX plus NSA group: Similar to the DOX group, these mice received DOX injections, but they also received 5 mg/kg of NSA i.p. daily for five days, starting two days before the DOX injection.

DOX and NSA doses were selected based on previous studies [9, 10].

### Serum and tissue preparation

On the fifth day, approximately two hours after the last dose administration, the mice were anesthetized using intraperitoneal injection of ketamine (80 mg/kg) and xylazine (10 mg/kg). Blood was collected by thoracotomy and direct cardiac puncture for biochemical analysis. The animals were then euthanized under anesthesia. The hearts were removed, weighed, washed twice with an ice-cold buffer solution, and divided into three pieces. The basal sections were utilized for the tissue homogenization procedure. The homogenates were centrifuged for fifteen minutes at a speed of 10,000 rpm at 4 ^o^C. To determine tissue markers, supernatants were used. The apical portions of the heart were preserved for histological examination in 10% neutral buffered formalin for 48 hours. The specimens were dehydrated in various alcohol concentrations, cleaned with xylene, embedded in paraffin, and hardened. The paraffin blocks were sliced into 5-µm slices and floated in a water bath. Floating slices were mounted on microscope slides, dried at 60 ºC for 20 minutes, stained with hematoxylin and eosin (H&E), and examined with light microscopy for histological alterations. Approximately 25 mg of cardiac tissue was homogenized for total ribonucleic acid (RNA) extraction in 500 µl of Trizol reagent. The serum and tissue homogenate were kept at -80 ºC until analysis.

### Enzyme immunoassay

Cardiac troponin I (cTnI) was used as a marker of acute myocardial injury and analyzed in serum with a mouse enzyme-linked immunosorbent assay (ELISA) kit (ELK Biotechnology, China). The homogenate of cardiac tissue was used to measure inflammatory markers (TNF-α and IL-1β), the antioxidant enzymes (glutathione peroxidase-4 (GPX-4) and heme oxygenase-1 (Hmox-1), pyroptosis related caspases (caspase-1) using their respective ELISA kits (ELK Biotechnology, China).

### Histopathological examination

Light microscopy was used to assess the severity of histopathological changes, as described by Bellingham and colleagues [11]. The histopathological changes were scored as follows: (I) myocardial fiber swelling and interstitial edema (1+), (II) myocardial cytoplasmic vacuolization/perinuclear vacuolization (2+), (III) myocardial fiber necrosis (3+), or no damage observed (0).

### Reverse Transcription-Polymerase Chain Reaction (RT-qPCR)

TRIzol reagent was utilized to extract total RNA from the cardiac sample (Thermo Fisher Scientific, USA), following kit instructions. 1µg of total RNA from each sample was utilized for first-strand cDNA synthesis using the Moloney Murine Leukemia Virus Reverse Transcriptase (M-MLV) kit Reverse Transcriptase kit (Bioneer, Korea). Gene expression of mice NF-κβ (F: GGACATTAAGCAGCTGACAGAAG and R: GTTTTAGAAGGGGCGGGACT) was determined using SYBR Green quantitative real-time PCR (SYBR Green kit, Bioneer, Korea). The relative expression levels for each sample were calculated after normalization against the reference gene (β-Actin) using the delta-delta Ct technique for comparing relative fold-expression changes.

### Statistical analysis

Data were displayed as means ± SD. One-way ANOVA followed by Fisher's Least Significant Difference (LSD) post hoc tests for multiple comparisons were used to assess statistical differences. Histopathological scores were analyzed using the Kruskal-Wallis test with pairwise analysis. The statistical analysis was conducted using the Statistical Package for the Social Sciences (SPSS) version 20 software, and differences were considered significant at p<0.05.

## RESULTS

### Serum cTnI levels

**Figure 1:**
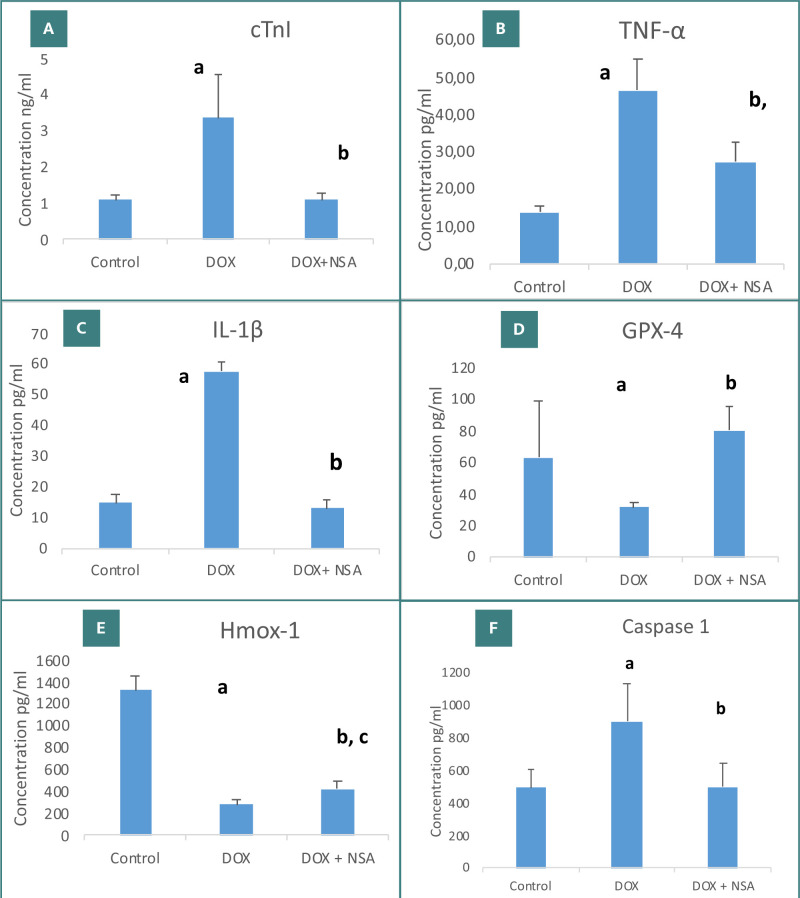
Effects of NSA on doxorubicin-induced cardiotoxicity in male mice; (A) cTnI, (B) TNF-α, (C) IL-1β, (D) GPX-4, (E) Hmox-1, and (F) caspase-1. Data is presented as mean ± SD and analyzed using one-way ANOVA followed by Fisher’s least significant difference (LSD) test. a p≤0.05 for DOX *vs*. control; b p≤0.05 for NSA plus DOX *vs*. DOX and c p≤0.05 for NSA plus DOX *vs*. control

DOX administration caused a significant increase in serum cTnI levels when compared to the control group (p<0.05). However, treatment with NSA resulted in a significant reduction (p<0.05) in serum cTnI concentration compared to the DOX group. Additionally, NSA administration nearly restored cTnI concentration to levels comparable to those of the control group (p>0.05), with no significant difference observed between the control and NSA-treated groups (Figure 1A).

### Cardiac tissue inflammatory markers (TNF-α and IL-1β)

Compared to the control group, the DOX group had significantly higher cardiac TNF-α and IL-1β levels (p<0.05). NSA treatment caused a significant reduction in both TNF-α and IL-1β levels compared to the DOX group (p<0.05). However, the TNF-α level remained significantly high in the NSA-treated group compared to the control (p<0.05), while the level of IL-1β was not significantly different (p>0.05) (Figure 1B-C).

### Cardiac antioxidant enzymes (GPX-4 and Hmox-1)

The DOX group showed a significant decrease in cardiac GPX-4 and Hmox-1 levels compared to the control group (p<0.05). However, treatment with NSA significantly increased GPX-4 and Hmox-1 concentrations compared to the DOX group (p<0.05). The administration of NSA did not restore Hmox-1 levels to those seen in the control group (p<0.05) (Figure 1 D-E).

### Cardiac caspase-1 levels

The DOX-treated group exhibited a significant increase in tissue caspase 1 levels compared to the control group (p<0.05). This effect was reversed by NSA treatment, resulting in a highly significant reduction in caspase-1 levels compared to the DOX group (p<0.05) (Figure 1F).

### NF-кβ gene expression in cardiac tissue

DOX caused a marked upregulation of NF-кβ gene expression compared to the control (mean fold changes=10.5±1.5, p<0.05). In contrast, NSA treatment led to a statistically significant downregulation of NF-кβ gene expression compared to the DOX-treated group (p<0.05). Furthermore, NSA treatment resulted in a mild and non-significant increase in gene expression levels compared to the control group, with a mean fold change of 1.5±0.75, p>0.05.

### Myocardial histopathological lesions

**Figure 2:**
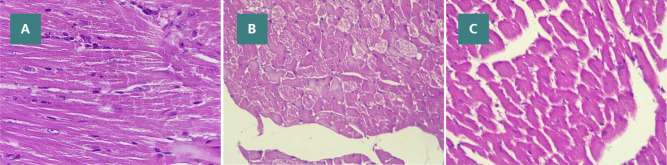
Microscopic images of myocardium sections at 400X magnification (A–C): (A) Control group - normal myocardium architecture, (B) DOX group - severe lesion with myocardial congestion, myofibrillar necrosis (black arrowhead) and vacuolization of the cytoplasm (yellow arrow) (C) DOX plus NSA group - mild lesion with no significant vacuolization or myofibrillar necrosis

In the control group, histopathological examination of the heart tissue revealed nearly normal morphological characteristics (Figure 2). However, DOX administration led to the development of severe myocardial lesions when compared to the control group, and this difference was statistically significant (p<0.05). These lesions were characterized by cytoplasmic vacuolization, multifocal congestion, and necrosis. In contrast, the NSA-treated group exhibited milder lesions with mild interstitial edema and no noticeable cytoplasmic vacuolization or myofibrillar necrosis. This difference in severity was significantly lower than that observed in the DOX group (p<0.05). Compared to the control group, no significant changes were observed in the severity score (p>0.05).

## Discussion

Doxorubicin is a powerful chemotherapeutic drug employed in treating various malignancies despite its association with a range of adverse effects, with cardiotoxicity being one of the most significant [12]. Currently, limited treatment options are available for doxorubicin-induced cardiotoxicity (DIC). Substantial efforts have been made in both fundamental and clinical research to identify preventive therapies [13]. The precise mechanisms underlying the development of DIC are not well understood. Myocardial cell damage is likely the first step in cardiotoxicity. DIC arises from a complex interaction of several mechanisms, including cytokine production, oxidative stress, iron-free radical generation, DNA damage, and myocyte membrane injuries. These mechanisms can lead to various forms of cell death, some of which are classified as regulated cell death (RCD) pathways [3]. Understanding the involvement of these pathways in DIC led us to explore necrosulfonamide as a potential treatment, given its ability to interfere with certain mechanisms associated with cardiotoxicity.

A single intraperitoneal injection of 20 mg/kg of doxorubicin-induced cardiotoxicity in this study, as evidenced by elevated serum cardiac troponin I levels, histopathological changes, increased inflammatory markers (TNF-α, IL-1β), decreased antioxidant enzymes (GPX-4, Hmox-1), and altered gene expression. One of the most trustworthy and widely used biomarkers for assessing DIC is cTnI [14, 15]. In this study, DOX administration caused cardiomyocyte injury, as shown by a substantial rise in the serum level of cTnI compared to controls. This aligns with several experimental studies that have described the response of cTnI to DOX [15, 16]. Treatment with NSA effectively mitigated the elevated serum cTnI levels, bringing them closer to normal levels. The cardioprotective potential of NSA has been previously explored in an ischemia-reperfusion (I/R) model of myocardial injury, where it demonstrated the ability to reduce infarct size and improve post-ischemic cardiac function in isolated Langendorff/perfused rat hearts [17]. Similarly, another experimental study revealed that NSA could modify post-resuscitation cardiac dysfunction through its anti-necroptosis and anti-pyroptosis effects [6].

### Effects on antioxidant enzymes, GPX-4, and Hmox-1

The administration of DOX led to a significant decrease in Hmox-1 concentration. This observation aligns with the findings of Gu *et al*. [18], who previously reported that DOX-induced damage to cardiomyocytes was accompanied by a reduction in Hmox-1 levels. However, Qin *et al*. [19] presented a contrasting result, showing a significant increase in Hmox-1 levels following DOX treatment. Treatment with NSA led to a significant increase in cardiac Hmox-1 levels, although it did not fully restore them to normal levels. The role of Hmox-1 was recently re-investigated, and both protective and harmful roles have been established [20-22]. These studies concluded that Hmox-1 is shown to convert heme to biliverdin/bilirubin, carbon-monoxide (CO), and Fe++ [23]. Thus, it has been proposed that Hmox-1 has cytoprotective antioxidant properties against different stress-related circumstances via its metabolites biliverdin/bilirubin, which can reduce lipid and protein peroxidation by scavenging ROS [24].

CO, another heme metabolite, has vasodilatory, anti-inflammatory, and anti-proliferative activities in various cell types [23, 25]. Moreover, it can collaborate with NF-κB to modify anti-apoptotic protein expression [26]. On the other hand, Fe++ derived from Hmox-1 metabolism is considered toxic, as it can interact with cellular components, leading to the generation of free radicals [22]. This can explain the pro-oxidative activity of Hmox-1 [20].

In our study, the role of Hmox-1 appears to be cardioprotective, aligning with the findings of Hull *et al*. [27], who highlighted that Hmox-1 contributes to cardiac protection, at least in part, by regulating mitochondrial autophagy. Regarding GPX-4, we observed a significant downregulation of GPX-4 in cardiac tissue following DOX administration, consistent with the results reported by Tadokoro *et al*. [28]. Additionally, Li *et al*. [29] also found that DIC was associated with the downregulation of GPX-4 and Hmox-1, and the restoration of these enzymes was linked to cardioprotection. The upregulation of GPX-4 levels observed with NSA treatment may be attributed to its potential antioxidant properties. NSA could exert its antioxidant effect by inhibiting MLKL1 phosphorylation and preserving mitochondrial integrity. This is particularly significant because mitochondrial dysfunction is known to be a major source of oxidative stress [30].

### Effects on inflammatory parameters

DOX exposure enhances oxidative stress and subsequently upregulates the expression of NF-κB gene. Consequently, more pro-inflammatory cytokines, including TNF-α and IL-1β, are released into the myocardial cells. NF-κB activation is the point at which numerous signal cascades, such as TNF-α, IL-1β, and other inflammatory cytokines, are controlled [31]. These findings are consistent with the observations in the current study, where DOX administration led to a significant upregulation of NF-κB gene expression, accompanied by a notable increase in tissue inflammatory biomarkers. However, NSA treatment effectively mitigated these effects, leading to a significant downregulation of NF-κB expression and a reduction in inflammatory biomarkers. These results align with previous studies highlighting the ability of NSA to suppress the release of pro-inflammatory cytokines through its inhibitory effects on pyroptosis and necroptosis [5-8, 32].

### Effects on caspases 1

The administration of DOX led to a significant activation of caspase 1, associated with pyroptosis. This finding aligns with Meng *et al*., who reported that DOX can induce pyroptosis in cardiomyocytes through the activation of caspase-1 and increase IL-1β secretion [33]. In the current study, NSA treatment caused significant inhibition of caspase-1 in cardiac tissue. A similar effect of NSA was reported previously but in a model of neuronal damage [34].

### Histopathological evaluation

Acute DOX injection resulted in significant cellular changes, including cytoplasmic vacuolization, myocardial fibers congestion, and myofibrillar necrosis. These cellular and structural alterations are consistent with earlier investigations on DIC [35, 36]. However, the histopathological analysis in the present study revealed that NSA mitigates the cardiac tissue damage induced by DOX. This was evidenced by a significant decrease in the severity score compared to the group treated with DOX alone.

## Conclusion

Our study demonstrated the cardioprotective effect of NSA against DIC, which is presumably mediated by its anti-pyroptosis, anti-necroptosis, and antioxidant effects.
